# Longitudinal plasma and urinary SP-D and sRAGE during BEP chemotherapy in germ cell tumours

**DOI:** 10.3389/fimmu.2026.1954888

**Published:** 2026-09-03

**Authors:** Katarina Rejlekova, Barbora Vlkova, Patrik Olah, Tomas Pokrivcak, Rateb Alzeer, Eva Escherova, Michal Chovanec, Peter Lesko, Jana Obertova, Zuzana Orszaghova, Patrik Palacka, Zuzana Sycova-Mila, Michal Mego, Peter Celec

**Affiliations:** 1 2nd Department of Oncology, Faculty of Medicine, Comenius University and National Cancer Institute, Bratislava, Slovakia; 2 Faculty of Medicine, Institute of Molecular Biomedicine, Comenius University, Bratislava, Slovakia; 3 Masaryk Memorial Cancer Institute, Brno, Czechia

**Keywords:** BEP chemotherapy, bleomycin, DLCO, germ cell tumours, neutropenia and/or febrile neutropenia, pulmonary toxicity, sRAGE, surfactant protein D

## Abstract

**Background:**

Bleomycin-induced pulmonary toxicity remains a significant complication of curative BEP chemotherapy for germ cell tumours, and no validated circulating biomarker enabling individual risk prediction or early detection of lung damage is currently available.

**Methods:**

This exploratory two-centre cohort study enrolled 60 patients with germ cell tumours receiving therapeutic three or four cycles of bleomycin, etoposide, and cisplatin (BEP) chemotherapy. A separate cohort of 18 patients with germ cell tumours receiving adjuvant single-cycle BEP was analysed independently. Plasma and urinary concentrations of surfactant protein D (SP-D) and soluble receptor for advanced glycation end-products (sRAGE) were evaluated before, during, and after BEP chemotherapy in relation to pulmonary toxicity, diffusing capacity of the lung for carbon monoxide (DLCO), pulmonary metastases, renal function, inflammatory indices, grade ≥3 neutropenia, febrile neutropenia, and other non-pulmonary toxicities.

**Results:**

Documented pulmonary toxicity developed in thirteen patients during or after BEP chemotherapy. Baseline plasma SP-D and sRAGE were not significantly associated with pulmonary toxicity (OR per doubling 0.60 [95% CI 0.27–1.37]; p=0.230 and OR 1.20 [95% CI 0.55–2.62]; p=0.640, respectively). The longitudinal increase of plasma SP-D during BEP chemotherapy was statistically significant (p=0.00018; n=30 with complete measurements), with a more pronounced increase associated with HRCT-confirmed pulmonary toxicity. Higher baseline urinary SP-D was significantly associated with lower post-treatment DLCO (Spearman coefficient rho=−0.609; p=0.001; FDR q=0.014). Plasma SP-D trajectories differed significantly according to the presence of pulmonary metastases (time × metastases interaction: p=0.012; adjusted p=0.027). Maximum plasma sRAGE was statistically significantly associated with neutropenia and/or febrile neutropenia during BEP chemotherapy (OR per doubling 12.13 [95% CI 2.59–56.93]; p=0.0016; FDR q=0.013), with this association remaining significant after correction for multiple testing.

**Conclusions:**

In patients with germ cell tumours receiving BEP chemotherapy, SP-D showed potential as a candidate monitoring biomarker of pulmonary injury during BEP chemotherapy. Greater SP-D increases were associated with HRCT-documented pulmonary toxicity in an exploratory analysis, whereas higher baseline urinary SP-D was significantly associated with impaired diffusing capacity of the lungs after treatment completion. Higher maximum plasma sRAGE was associated with neutropenia and/or febrile neutropenia. These results support prospective validation of compartment-specific monitoring of SP-D and sRAGE during BEP chemotherapy.

## 1 Introduction

BEP chemotherapy represents the cornerstone of curative treatment for metastatic germ cell tumours. As the majority of patients are young with an expectation of long-term survival, prevention of serious pulmonary morbidity is among the foremost clinical priorities (1–4).

Clinically overt bleomycin-induced pneumonitis occurs in approximately 10–20% of treated patients, with fatal outcomes reported in 1–3%; subclinical lung injury — manifest as long-term impairment of diffusing capacity and persistent radiological abnormalities — is substantially more frequent and has been documented years after treatment completion (4, 6). Bleomycin pulmonary toxicity is characterised by marked interindividual variability and does not correlate consistently with cumulative dose, with clinically significant toxicity occurring even in patients without identifiable risk factors (4, 6). The underlying mechanism is immune-mediated: bleomycin generates iron-dependent reactive oxygen species that cause DNA strand breaks and oxidative injury to alveolar epithelial and endothelial cells, triggering the release of damage-associated molecular patterns (DAMPs), dysregulated innate immune activation, and profibrotic signalling (6). The low activity of bleomycin hydrolase — the principal enzyme responsible for bleomycin inactivation — in lung tissue provides a biologic basis for the characteristic susceptibility of the pulmonary parenchyma to bleomycin-induced injury, and positions bleomycin-induced pulmonary toxicity as a model of immune-mediated chemotherapy toxicity (4, 6).

Clinical assessment of bleomycin pulmonary toxicity is based on symptoms, imaging studies, pulmonary function tests, cumulative exposure, age, and renal function. However, no validated biomarker exists that can reliably identify at-risk patients prior to treatment or detect alveolar damage early enough to inform the decision to omit bleomycin (1–3, 5, 6). Early clinical manifestations are often non-specific and may appear only after substantial alveolar injury has occurred, underscoring the need for circulating biomarkers that capture the onset of lung injury at the molecular level.

SP-D is a glycoprotein produced predominantly by type II alveolar epithelial cells that regulates surfactant homeostasis and modulates innate pulmonary immunity; during lung injury, disruption of the alveolar-capillary barrier permits leakage of SP-D into the systemic circulation, providing a mechanistic basis for its measurement as a circulating biomarker of alveolar epithelial injury (7–9). sRAGE is the soluble ectodomain of RAGE, a receptor preferentially expressed by type I alveolar cells, and serves as a systemic indicator of alveolar epithelial integrity; elevated plasma sRAGE concentrations are independently associated with worse clinical outcomes in acute respiratory distress syndrome (ARDS) (10, 11). Urinary concentrations were measured alongside plasma, as renal filtration of circulating proteins and potential direct tubular involvement may allow different compartments to capture distinct aspects of alveolar injury and systemic response. Despite the biological plausibility of both markers as circulating indicators of bleomycin-induced alveolar damage, their longitudinal kinetics during BEP chemotherapy, predictive value for clinically relevant endpoints, and the relationship between plasma and urinary concentrations in this population have not been systematically characterised (7–9, 12).

We evaluated baseline and longitudinal plasma and urinary SP-D and sRAGE concentrations during therapeutic BEP, with adjuvant single-cycle BEP analysed separately. Patients with germ cell tumours represent an ideal study population for translational biomarker research given their young age, curative treatment intent, protocolised chemotherapy delivery, and the clinical importance of avoiding unnecessary bleomycin-related pulmonary morbidity. We assessed pulmonary toxicity, DLCO, pulmonary metastases, renal and inflammatory factors, treatment response, and the concordance between plasma and urinary values.

## 2 Materials and methods

### 2.1 Study design and population

The total study population comprised 78 patients. The therapeutic cohort consisted of 60 patients with germ cell tumours treated with three or four therapeutic cycles of BEP at the National Cancer Institute in Bratislava and Masaryk Memorial Cancer Institute in Brno. The adjuvant cohort comprised 18 patients receiving single-cycle BEP, who were excluded from the main therapeutic cohort and analysed separately. Three patients died during the first cycle of chemotherapy due to septic shock (serious adverse event, SAE) and were excluded from longitudinal and post-treatment analyses.

### 2.2 Biological sample collection, processing, and storage

Peripheral blood samples were collected into ethylenediaminetetraacetic acid (EDTA)-containing tubes. Plasma was separated by centrifugation and stored at −80 °C until analysis. For patients contributing longitudinal biomarker data, samples were collected at predefined time points: before the first BEP cycle (baseline); on Day 22 of each preceding cycle—that is, immediately before bleomycin administration of each subsequent cycle—and four weeks after completion of BEP. This standardised schedule was specified *a priori* to ensure a consistent interval between bleomycin exposure and biomarker measurement, thereby minimising inter-patient variability in sampling timing. Thirty patients from the therapeutic cohort had complete longitudinal plasma measurements at all applicable predefined time points. Urine samples were collected at the respective available study control visits and stored frozen until biomarker determination. Prior to analysis, samples and all kit components were equilibrated to room temperature. Plasma and urinary concentrations of soluble receptor for advanced glycation end-products (sRAGE) and surfactant protein D (SP-D) were determined using commercially available sandwich ELISA kits (BioVendor – Laboratorni medicína a.s., Brno, Czech Republic) according to the manufacturer’s instructions. Standards, controls, and patient samples were analysed on antibody-coated microtitre plates and concentrations were calculated from the respective calibration curves.

### 2.3 Laboratory determination of sRAGE

Sample preparation. EDTA plasma samples were thawed immediately before analysis and diluted 1:3 using the supplied Dilution Buffer. Urine samples were analysed undiluted.

Reagent preparation. All kit components and samples were equilibrated to 20–25 °C before commencing analysis. The wash solution was prepared by diluting the supplied concentrate with distilled water. The lyophilised sRAGE standard was reconstituted and serially diluted to generate a calibration curve.

Sample incubation. 100 µl of each standard, control, 3-fold diluted EDTA plasma, or undiluted urine was pipetted into wells of a microtitre plate coated with polyclonal anti-sRAGE antibody. The plate was incubated for 2 hours at room temperature on an orbital shaker at approximately 300 rpm.

Washing and detection. Well contents were aspirated and the plate was washed five times with 350 µl of wash solution per well. After the final wash, the plate was blotted dry on absorbent paper. Subsequently, 100 µl of biotinylated detection antibody was added to each well, with incubation for 1 hour at 25 °C on an orbital shaker at 300 rpm. The washing cycle was then repeated. 100 µl of streptavidin-horseradish peroxidase conjugate was added to each well and the plate was incubated for 30 minutes at 25 °C on an orbital shaker at 300 rpm, followed by a further washing cycle.

Colour development and measurement. 100 µl of substrate solution was added to each well and the plate was incubated for 15 minutes at 25 °C in the dark. The reaction was stopped by the addition of 100 µl of stop solution. Absorbance was measured immediately at 450 nm and sRAGE concentrations were calculated from the calibration curve.

### 2.4 Laboratory determination of SP-D

Sample preparation. EDTA plasma samples were thawed immediately before analysis and diluted 1:11 using the supplied Dilution Buffer. Urine samples were analysed undiluted.

Reagent preparation. All reagents and samples were equilibrated to room temperature before analysis. The wash solution was prepared by diluting the concentrated wash buffer. The lyophilised SP-D standard was reconstituted and serially diluted to generate a calibration curve.

Sample incubation. 100 µl of each standard, control, 11-fold diluted EDTA plasma, or undiluted urine was pipetted into wells of a microtitre plate coated with monoclonal anti-SP-D antibody. The plate was incubated for 2 hours at 25 °C on an orbital shaker at 300 rpm.

Washing and detection. Well contents were aspirated and the plate was washed five times with 350 µl of wash solution per well. After the final wash, the plate was blotted dry on absorbent paper. 100 µl of biotinylated detection antibody was added to each well, with incubation for 1 hour at 25 °C on an orbital shaker at 300 rpm. The washing cycle was then repeated. 100 µl of streptavidin-horseradish peroxidase conjugate was added to each well and the plate was incubated for 1 hour at 25 °C on an orbital shaker at 300 rpm, followed by a further washing cycle.

Colour development and measurement. 100 µl of substrate solution was added to each well and the plate was incubated for 15 minutes at 25 °C in the dark. The reaction was stopped by the addition of 100 µl of stop solution. Absorbance was measured immediately at 450 nm and SP-D concentrations were calculated from the calibration curve.

For both assays, when more than one measurement was available for the same patient at the same time point, the arithmetic mean was used in the statistical analysis and the number of source measurements was recorded.

### 2.5 Study endpoints and outcome definitions

The primary clinical endpoint was documented pulmonary toxicity, defined as the presence of compatible respiratory symptoms and/or a positive high-resolution computed tomography (HRCT) finding in the clinical context of BEP chemotherapy, assessed in accordance with established diagnostic criteria (3, 6, 13). All patients underwent routine post-treatment computed tomography of the thorax, abdomen, and pelvis as part of standard oncological restaging; HRCT was additionally performed in patients with compatible respiratory symptoms and in asymptomatic patients in whom the restaging CT identified pulmonary changes potentially consistent with bleomycin-induced injury. Patients in whom HRCT was not performed were classified as negative for the HRCT-defined endpoint. Compatible respiratory symptoms comprised new or worsening exertional dyspnoea, non-productive cough, or reduced exercise tolerance; fever and hypoxaemia were considered indicative of more advanced inflammatory involvement (6). A positive HRCT finding was defined as the presence of any of the following in a distribution consistent with bleomycin-induced pulmonary injury: ground-glass opacities, subpleural reticulations, consolidations, traction bronchiectasis, or linear infiltrates, predominantly in a bilateral, lower-lobe, and dorsobasal subpleural distribution (3, 6, 13). As bleomycin-induced pulmonary toxicity frequently presents as asymptomatic radiological abnormalities, HRCT findings were given diagnostic primacy over symptoms alone (6). Pre-specified secondary clinical endpoints comprised: (i) symptomatic pulmonary toxicity, defined as respiratory symptoms meeting the above criteria without HRCT confirmation; (ii) strict HRCT-documented pulmonary toxicity, defined as a positive HRCT finding irrespective of the presence of symptoms; (iii) post-treatment diffusing capacity of the lungs for carbon monoxide (DLCO), as a measure of functional pulmonary impairment; and (iv) haematological toxicity, defined as grade ≥3 neutropenia or febrile neutropenia occurring during any cycle of BEP chemotherapy. The primary biomarker analysis evaluated whether longitudinal plasma SP-D dynamics discriminated patients with HRCT-defined pulmonary toxicity from those without, assessed using the last available percentage change from baseline and receiver operating characteristic (ROC) analysis. Secondary and exploratory biomarker analyses included: longitudinal plasma sRAGE; urinary SP-D and sRAGE at available time points; the predictive value of baseline biomarker concentrations for clinical endpoints; the effect of pulmonary metastasis status on biomarker kinetics; and the association of maximum biomarker concentrations with haematological outcomes. Assessed clinical covariates included age, smoking history, haemoglobin, creatinine, estimated glomerular filtration rate (eGFR), cumulative bleomycin dose, granulocyte colony-stimulating factor (G-CSF) use, neutrophil-to-lymphocyte ratio (NLR), platelet-to-lymphocyte ratio (PLR), systemic immune-inflammation index (SII) (14), IGCCCG risk classification, and treatment response.

### 2.6 Statistical analysis

Continuous variables are reported as median and interquartile range, categorical variables as counts and percentages. The Mann-Whitney U test was used to compare independent groups, the Wilcoxon signed-rank test for paired changes, Fisher’s exact test for binary associations, and Spearman’s rank correlation for continuous associations. In logistic regression, biomarkers were log2-transformed, so the OR expresses the effect of a doubling of the biomarker value. Effect estimates are reported with 95% confidence intervals. Longitudinal models included time, group, and time × group interaction with a patient-level random intercept; for singular models, ordinary least squares with robust standard errors clustered by patient was used. The false discovery rate was controlled using the Benjamini–Hochberg method. Analyses were performed using available cases for the variables required for each analysis. Longitudinal models included all available repeated measurements (15, 16).

## 3 Results

The primary statistically significant findings emerging from this study were: (i) a significant cohort-level longitudinal increase in plasma SP-D during BEP chemotherapy, with exploratory evidence that the amplitude of increase distinguished patients who developed HRCT-defined pulmonary toxicity from those who did not (Sections 3.4 and 3.5); (ii) a significant association between higher baseline urinary SP-D and lower post-treatment diffusing capacity of the lungs for carbon monoxide (DLCO), representing the finding most robustly supported after correction for multiple testing (Section 3.7); and (iii) an association between maximum plasma sRAGE and the development of neutropenia and/or febrile neutropenia during BEP (Section 3.6). Baseline plasma biomarker concentrations did not predict the development of pulmonary toxicity (Section 3.3). Findings from the separate adjuvant cohort and the 3×BEP versus 4×BEP comparison are presented in Sections 3.9 and 3.10 and are regarded as exploratory.

### 3.1 Characteristics of the main cohort

**Figure 1:**
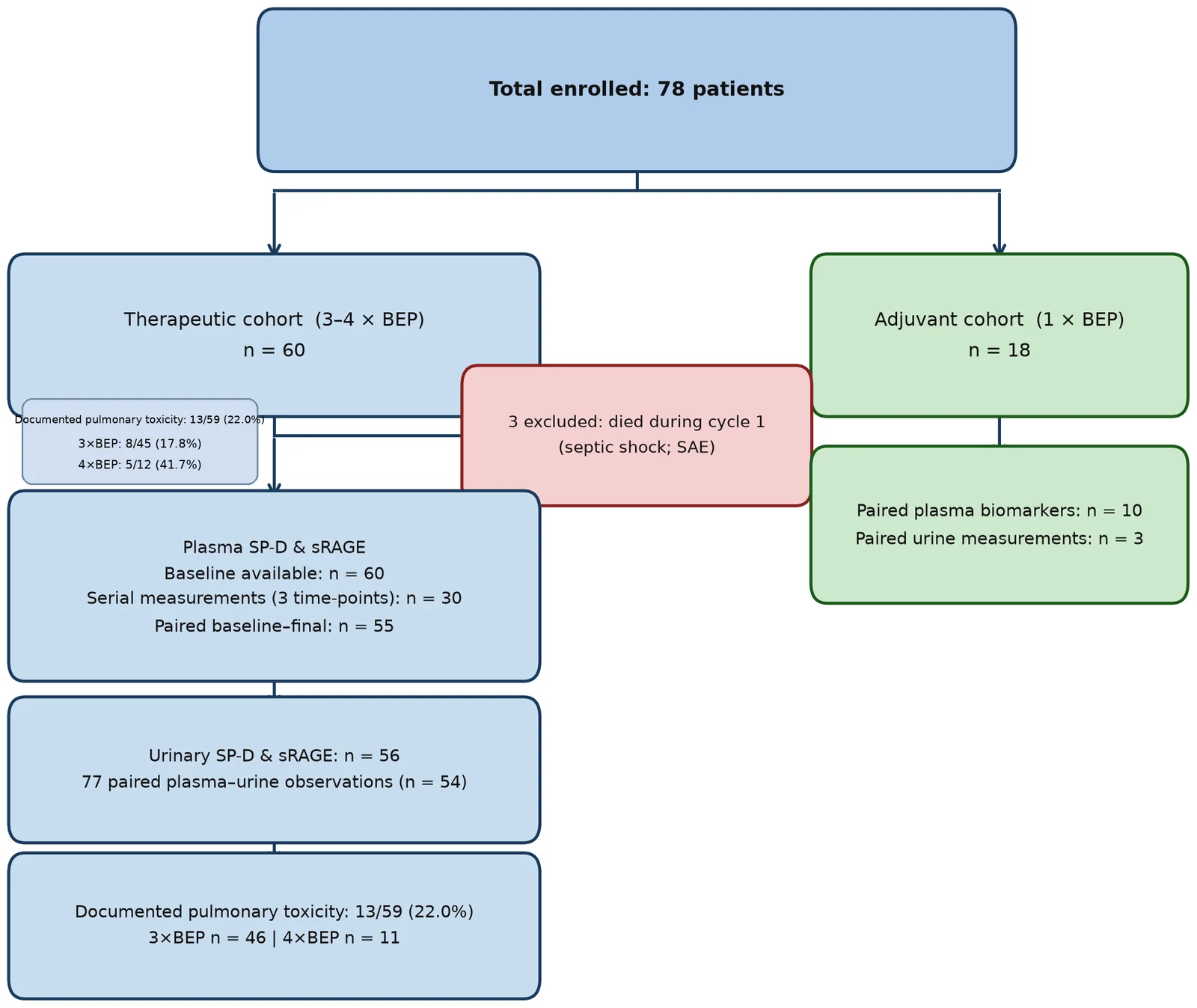
Study cohort and availability of plasma, urinary, toxicity, and paired inter-compartment data.

The therapeutic cohort comprised 60 patients: 46 patients were treated with three cycles of BEP and 11 patients with four cycles of BEP. Three patients enrolled in the therapeutic cohort died during the first cycle of chemotherapy due to septic shock (serious adverse event, SAE) and were excluded from longitudinal and post-treatment analyses. Baseline plasma SP-D and sRAGE were available for 60 patients. Documented pulmonary toxicity occurred in thirteen patients. Urinary data were available for 56 patients and 77 paired plasma-urine observations were available from 54 patients (**Table 1**, **Figure 1**). Detailed characteristics of the entire therapeutic cohort including the 3×BEP and 4×BEP subgroups are provided in **Supplementary Table 1**. IGCCCG classification was applicable to 42 patients with metastatic disease; the distribution was good-risk in 31 (73.8%), intermediate-risk in 6 (14.3%), and poor-risk in 5 (11.9%). IGCCCG classification was not significantly associated with HRCT-defined pulmonary toxicity (chi-square p=0.397), baseline plasma SP-D (Kruskal-Wallis p=0.174), or baseline plasma sRAGE (p=0.794). A borderline significant difference in the longitudinal plasma SP-D trajectory was observed across IGCCCG categories (Kruskal-Wallis p=0.046), driven by a numerically larger median SP-D increase in intermediate-risk patients (+89.3 ng/mL) compared with good-risk (+29.3 ng/mL) and poor-risk (+37.4 ng/mL) patients. IGCCCG classification was significantly associated with clinical symptom-based pulmonary toxicity (chi-square p=0.002): symptoms consistent with pulmonary toxicity were reported in 60.0% of intermediate-risk patients, compared with 6.5% of good-risk and 0% of poor-risk patients; these results should be interpreted cautiously given the limited numbers in the non-good-risk groups.

**Table 1:** Baseline and clinical characteristics.

Characteristic	All patients	Without toxicity	Pulmonary toxicity	P
Age, years	36.0 (32.0-41.0)	37.0 (32.0-41.8)	36.0 (32.0-37.0)	0.2164
Age ≥30 years	46/59 (78.0%)	36/46 (78.3%)	10/13 (76.9%)	1.0000
Smoking history	28/56 (50.0%)	24/43 (55.8%)	4/13 (30.8%)	0.2047
Pulmonary metastases	12/55 (21.8%)	10/42 (23.8%)	2/13 (15.4%)	0.7093
Haemoglobin, g/L	150.0 (139.0-156.0)	152.5 (142.0-157.2)	117.0 (110.0-149.0)	0.0015
Creatinine, µmol/L	80.3 (73.9-91.5)	81.2 (75.0-88.7)	77.3 (71.2-93.7)	0.7798
eGFR, mL/min	108.9 (90.5-116.2)	108.3 (95.5-114.9)	109.5 (80.7-120.6)	0.8888
Baseline DLCO, % predicted	95.0 (85.5-101.5)	95.0 (87.8-101.8)	98.0 (78.0-101.0)	0.5454
Post-treatment DLCO, % predicted	96.0 (83.2-103.2)	102.0 (94.0-105.0)	84.0 (75.0-87.5)	0.0063
DLCO change, percentage points	-4.0 (-12.8-2.5)	-3.0 (-10.0-4.0)	-10.0 (-17.0--4.0)	0.0971
NLR	2.5 (1.9-3.8)	2.2 (1.8-3.4)	3.1 (2.5-4.6)	0.0763
PLR	141.2 (115.0-210.3)	133.6 (113.4-175.7)	200.6 (167.5-293.6)	0.0019
SII	570.6 (404.5-885.6)	527.6 (375.9-821.2)	859.2 (583.7-1117.1)	0.0517
Cumulative bleomycin dose, mg	270.0 (270.0-360.0)	270.0 (270.0-337.5)	270.0 (270.0-360.0)	0.8187
G-CSF use	57/57 (100.0%)	44/44 (100.0%)	13/13 (100.0%)	

### 3.2 Clinical factors associated with pulmonary toxicity

Patients with pulmonary toxicity had lower baseline haemoglobin (p=0.0015), higher PLR (p=0.0019), and lower post-treatment DLCO (p=0.0063). Median baseline haemoglobin was substantially lower in patients with documented pulmonary toxicity compared with those without (117.0 [IQR 110.0–149.0] vs 152.5 [IQR 142.0–157.2] g/L), suggesting that lower pre-treatment haemoglobin may be associated with higher risk of pulmonary toxicity; the clinical significance of this observation requires confirmation in larger studies. Higher baseline PLR (200.6 [IQR 167.5–293.6] vs 133.6 [IQR 113.4–175.7]) was observed in patients who developed pulmonary toxicity, consistent with a possible relationship between systemic inflammatory status and pulmonary toxicity. Lower post-treatment DLCO in patients with documented toxicity (84.0 [IQR 75.0–87.5] vs 102.0 [IQR 94.0–105.0] % predicted) was consistent with greater functional pulmonary impairment in patients with documented toxicity. NLR and SII were numerically higher in the toxicity group but did not reach statistical significance (p=0.076 and p=0.052, respectively). PLR showed the strongest nominal association among the evaluated inflammatory indices in this dataset.

### 3.3 Predictive value of baseline plasma biomarker concentrations

Baseline SP-D and sRAGE did not predict pulmonary toxicity: OR per doubling was 0.60 (95% CI 0.27–1.37; p=0.230) and 1.20 (95% CI 0.55–2.62; p=0.640), respectively. An OR below 1 for SP-D indicates that higher pre-treatment SP-D was not associated with increased toxicity risk, which may reflect inter-individual variability in baseline alveolar physiology rather than a protective effect. No statistically significant association was detected between baseline sRAGE and pulmonary toxicity in this cohort. These findings are consistent with the established principle that bleomycin-induced alveolar damage is a dynamic, cumulative process that unfolds during treatment and is not reliably reflected by pre-treatment biomarker concentrations. The results reinforce that single pre-treatment measurements of SP-D or sRAGE are insufficient to identify patients who will develop pulmonary toxicity, and that longitudinal biomarker changes showed evidence of discrimination in exploratory analyses, whereas baseline concentrations did not show statistically significant associations with pulmonary toxicity.

### 3.4 Plasma SP-D dynamics during BEP chemotherapy: significant elevation and correlation with HRCT-defined pulmonary toxicity

**Figure 2:**
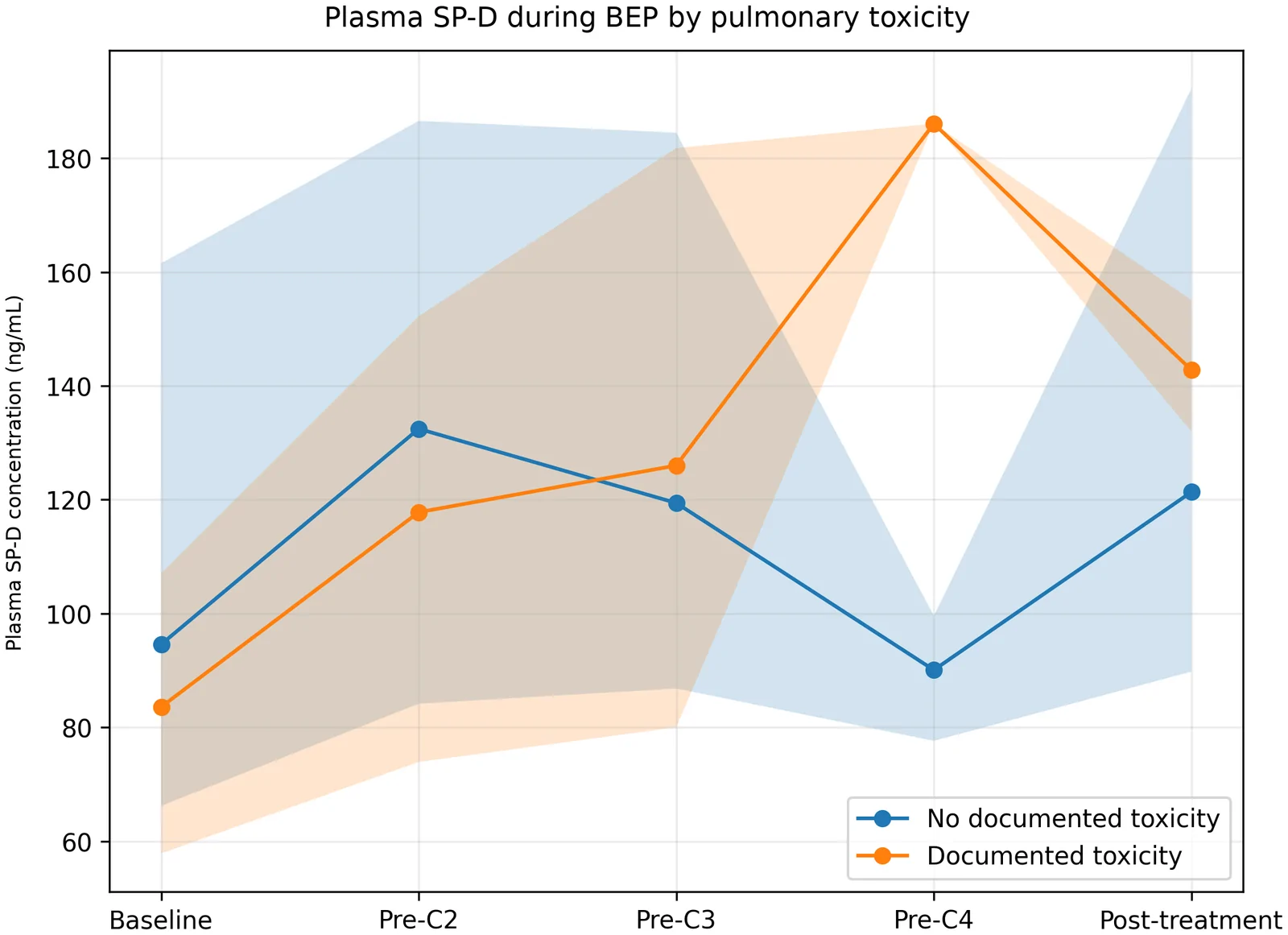
Median plasma SP-D concentration during BEP according to documented pulmonary toxicity; shaded areas represent interquartile ranges.

**Figure 3:**
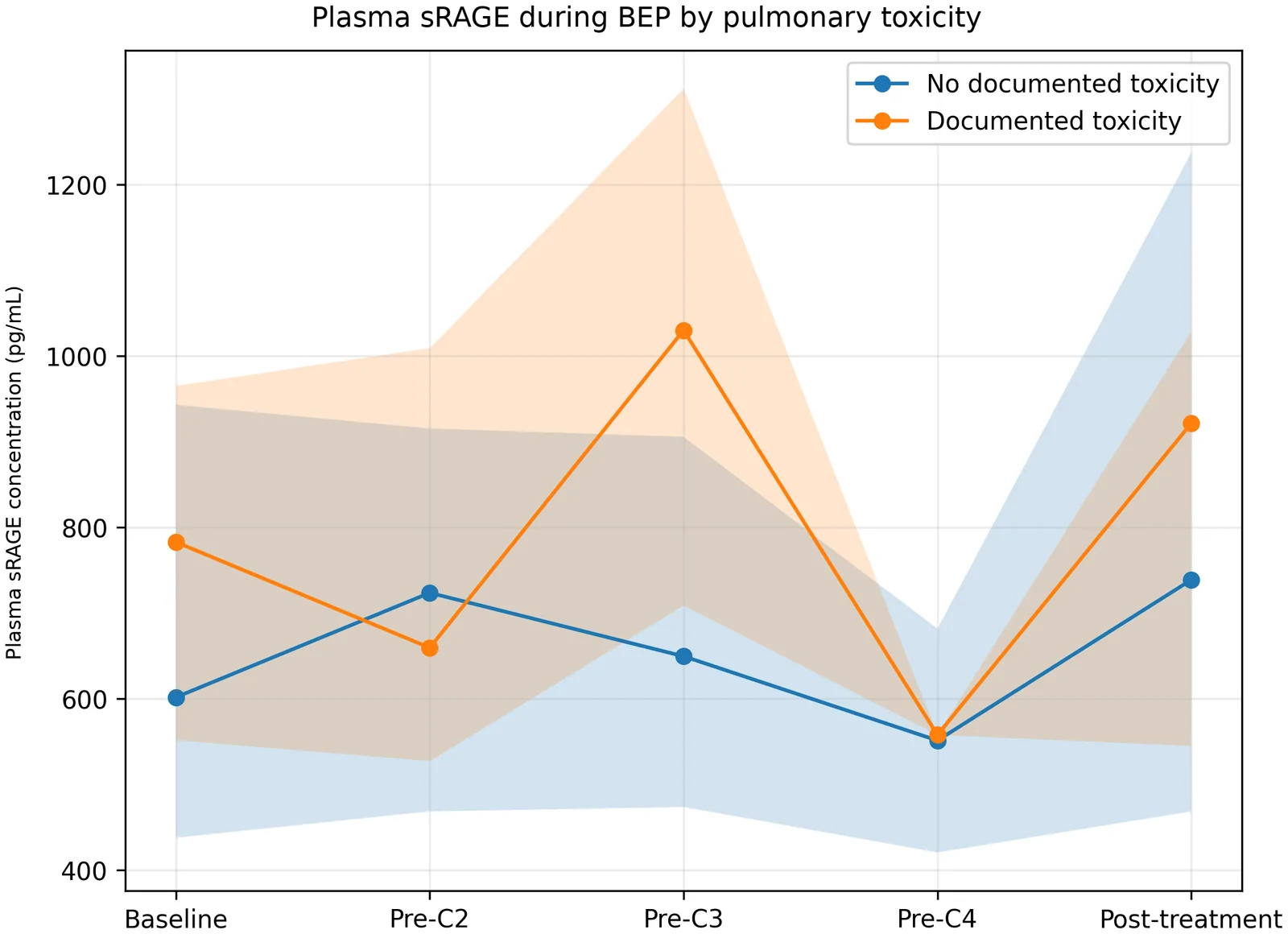
Median plasma sRAGE concentration during BEP according to documented pulmonary toxicity; shaded areas represent interquartile ranges.

A more pronounced longitudinal increase in plasma SP-D was associated with HRCT-defined pulmonary toxicity. Median plasma SP-D rose from 92.4 ng/mL before the first cycle to 134.9 ng/mL before the second cycle and 123.7 ng/mL before the third cycle. The last available percentage increase in SP-D was higher in patients with HRCT toxicity (p=0.010), although the association did not retain statistical significance after FDR correction (q=0.228). Among 55 patients with available baseline and final measurements, the median individual increase in SP-D was 44.2 ng/mL (IQR 2.8–84.2; p<0.001). The overall time × toxicity interaction was not significant for either SP-D or sRAGE. To address the potential confounding influence of pulmonary metastases on the SP-D–toxicity association, the relationship between the last available percentage SP-D increase and HRCT-defined pulmonary toxicity was re-evaluated after accounting for pulmonary metastasis status. In logistic regression adjusting for pulmonary metastasis status (n=26, HRCT-positive n=7), the SP-D percentage increase remained directionally associated with toxicity (OR 1.007 per 1% increase; 95% CI 0.997–1.016; p=0.156). In stratified analyses within patients without pulmonary metastases (HRCT-positive n=5; median SP-D increase +112.0% vs +39.1%; p=0.107) and within patients with pulmonary metastases (HRCT-positive n=2; median SP-D increase +153.8% vs +7.6%; p=0.095), the direction of effect was consistent in both strata—patients with HRCT-defined toxicity showed greater SP-D increases than those without, regardless of pulmonary metastasis status. Formal statistical significance was not achieved in the adjusted and stratified analyses, which may in part reflect the limited number of HRCT-positive events. In ROC analysis (n=26; HRCT-positive n=7), the SP-D percentage increase discriminated HRCT-defined toxicity with an AUC of 0.827 (95% CI 0.637–0.967); at the optimal Youden threshold of >79.7% increase, sensitivity was 85.7% and specificity 84.2% (**Figures 2**, **3**).

### 3.5 Pulmonary metastases and biomarker trajectories

**Figure 4:**
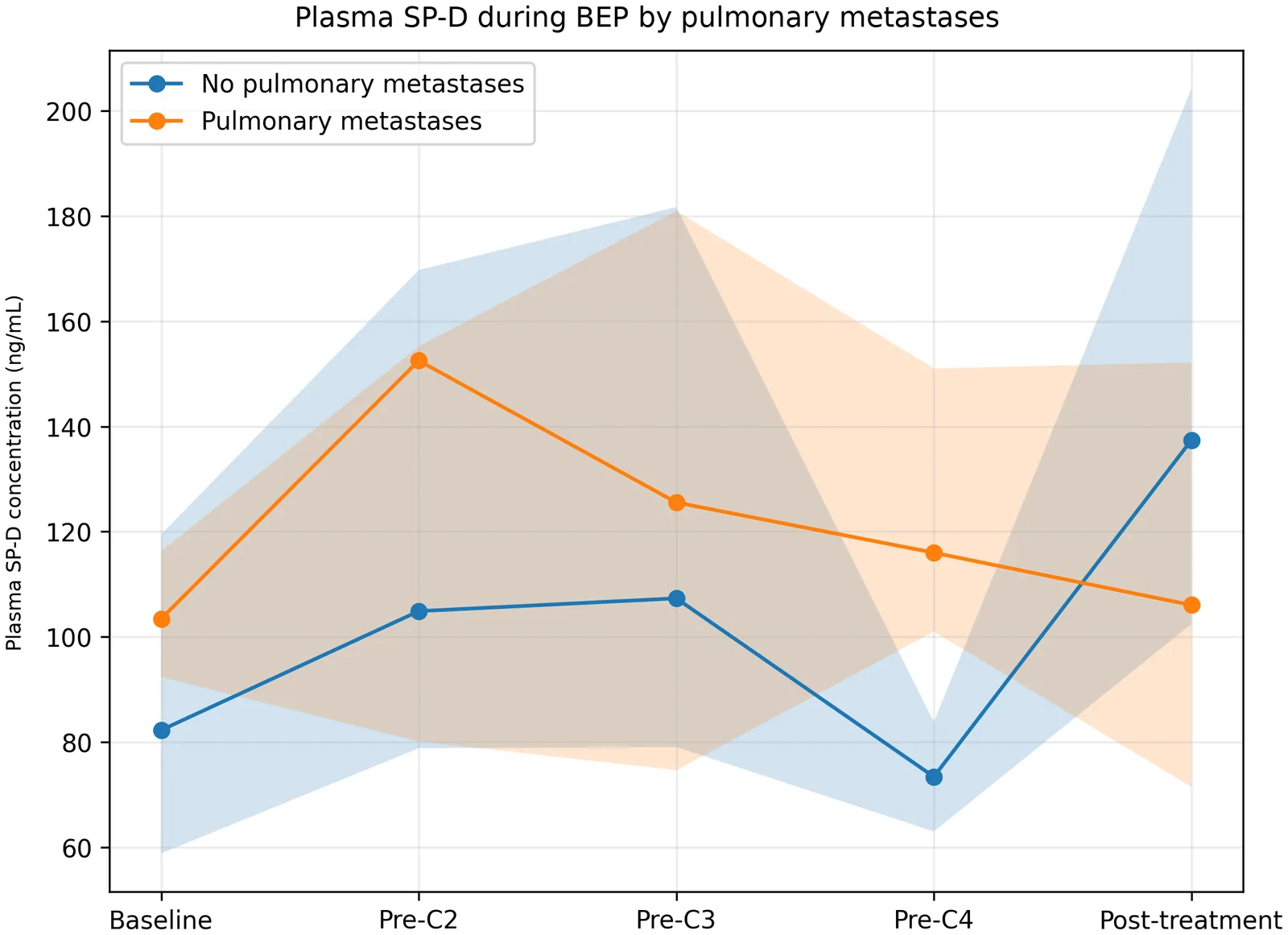
Median plasma SP-D concentration during BEP according to the presence of pulmonary metastases. Time × metastases interaction was significant in the unadjusted model (p=0.012) and in the adjusted model (p=0.027).

To evaluate whether pulmonary metastasis status affects the discriminatory capacity of SP-D for pulmonary toxicity, ROC analysis was performed stratified by baseline pulmonary metastasis status. Among patients without pulmonary metastases (n=19; HRCT-positive n=5), AUC was 0.757 (95% CI 0.529–0.938), with sensitivity 100% and specificity 64.3% at the optimal threshold of >52.0% SP-D increase. Among patients with pulmonary metastases (n=7; HRCT-positive n=2), perfect rank-order discrimination was observed: both HRCT-positive patients showed markedly greater SP-D increases (+91.0% and +216.6%) than all five HRCT-negative patients in this stratum (median +7.6%; range −49.3% to +61.0%). Strikingly, the median SP-D increase in HRCT-positive patients with pulmonary metastases (+153.8%) was higher than in HRCT-positive patients without metastases (+112.0%), indicating that bleomycin-induced alveolar injury generates a SP-D signal that is not attenuated by concurrent metastasis regression. These results should be interpreted cautiously given the very small subgroup sizes (n=2 HRCT-positive patients with pulmonary metastases).SP-D increased less over time in patients with pulmonary metastases. The time × metastases interaction was significant in the unadjusted model (p=0.012) and after adjustment for age, smoking, haemoglobin, and eGFR (p=0.027) (**Figure 4**). Pulmonary metastases were not associated with the occurrence of toxicity (Fisher’s test p=0.709). A plausible biological explanation is that tumour regression under chemotherapy may alter the pulmonary epithelial environment in a way that partially offsets the bleomycin-driven rise in circulating SP-D, as resolving metastatic deposits may restore rather than further disrupt alveolar barrier integrity. Pulmonary metastasis status was not significantly associated with toxicity in this cohort. These findings underscore the importance of stratifying longitudinal biomarker analyses by pulmonary metastasis status in future studies, to avoid misattributing metastasis-driven SP-D changes to bleomycin toxicity.

### 3.6 Associations with renal and pulmonary function

Baseline plasma sRAGE correlated with creatinine (rho=0.455; p=0.0028) and negatively with eGFR (rho=−0.396; p=0.010). The percentage increase in SP-D after treatment correlated with DLCO change (rho=−0.648; p=0.012; q=0.114). Maximum plasma sRAGE was higher in patients with neutropenia and/or febrile neutropenia compared with patients without this toxicity (1, 347.8 versus 845.1 pg/mL; p=0.00023) (**Table 2**).

**Table 2:** Key findings for plasma biomarker concentrations.

Predictor	Endpoint	N	Effect	p	Method
Baseline SP-D	Any pulmonary toxicity	60	OR 0.60 (95% CI 0.27-1.37)	0.2300	Logistic regression; OR per doubling
Baseline sRAGE	Any pulmonary toxicity	60	OR 1.20 (95% CI 0.55-2.62)	0.6400	Logistic regression; OR per doubling
Last percentage SP-D change	HRCT toxicity	26	Higher in HRCT toxicity	0.0100	Mann-Whitney U test; FDR q=0.228
ROC analysis: last percentage SP-D increase	HRCT-defined pulmonary toxicity	26	AUC 0.827 (95% CI 0.637–0.967); Youden threshold >79.7%: sensitivity 85.7%, specificity 84.2%	0.0100	ROC curve; FDR q=0.228
Time × toxicity interaction, SP-D	Longitudinal trajectory	187	Interaction	0.1040	Linear mixed model
Time × pulmonary metastases interaction, SP-D	Longitudinal trajectory	148	Interaction	0.0120	Mixed model; adjusted p=0.027
Baseline sRAGE	Creatinine	41	rho=0.455	0.0028	Spearman; FDR in plasma family q=0.036
Percentage SP-D change post-treatment	DLCO change	14	rho=-0.648	0.0120	Spearman; FDR q=0.114
Maximum plasma sRAGE	Neutropenia and/or febrile neutropenia	Available cases	OR 12.13 (95% CI 2.59-56.93)	0.0016	Logistic regression; OR per doubling; FDR q=0.013

### 3.7 Urinary biomarker concentrations: higher baseline urinary SP-D is associated with lower post-treatment diffusing capacity

**Figure 5:**
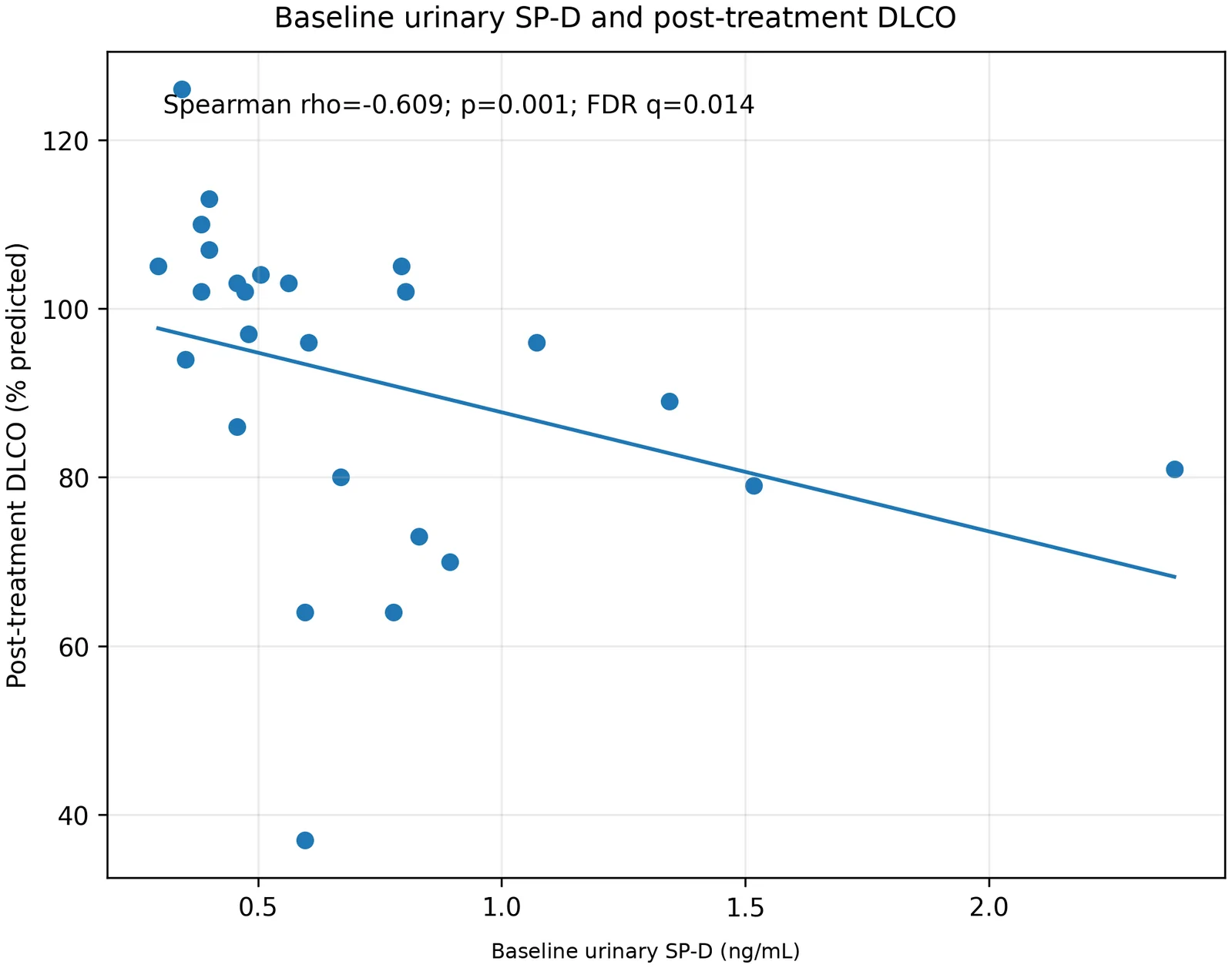
Baseline urinary SP-D concentration and post-treatment DLCO (Spearman rho=−0.609; p=0.001; FDR q=0.014).

Baseline urinary SP-D was associated with lower baseline DLCO (rho=-0.530; p=0.0037) and lower post-treatment DLCO (rho=-0.609; p=0.0010; q=0.014) (**Table 3**, **Figure 5**). As baseline urinary SP-D was also correlated with baseline DLCO (ρ=−0.530; p=0.0037), a multivariable model adjusting for baseline DLCO would be required to establish a significant association with post-treatment pulmonary function. Maximum urinary sRAGE was nominally higher in patients with HRCT toxicity (p=0.0159; q=0.351). Urinary sRAGE showed a nominally significant time × toxicity interaction (p=0.019) in the model with robust standard errors clustered by patient.

**Table 3:** Key findings for urinary biomarker concentrations and their relationship to plasma concentrations.

Predictor/comparison	Endpoint	N	Effect	p	FDR q	Method
Baseline urinary SP-D	Post-treatment DLCO	26	rho=-0.609	0.0010	0.0140	Spearman
Baseline urinary SP-D	Baseline DLCO	28	rho=-0.530	0.0037	0.0390	Spearman
Maximum urinary sRAGE	HRCT toxicity	35	Higher in HRCT toxicity	0.0159	0.3510	Mann-Whitney U
Time × toxicity interaction, urinary sRAGE	Longitudinal trajectory	95	Interaction	0.0190		OLS with clustered standard errors
Baseline plasma SP-D vs urinary SP-D	Baseline values of the same patient	46	rho=-0.062	0.6840		Spearman
Baseline plasma sRAGE vs urinary sRAGE	Baseline values of the same patient	46	rho=-0.140	0.3540		Spearman
All time points: plasma SP-D vs urinary SP-D	Paired repeated measurements	72	rho=-0.233	0.0490	0.3940	Spearman; clustered model p=0.046

### 3.8 Concordance between plasma and urinary biomarker concentrations

**Figure 6:**
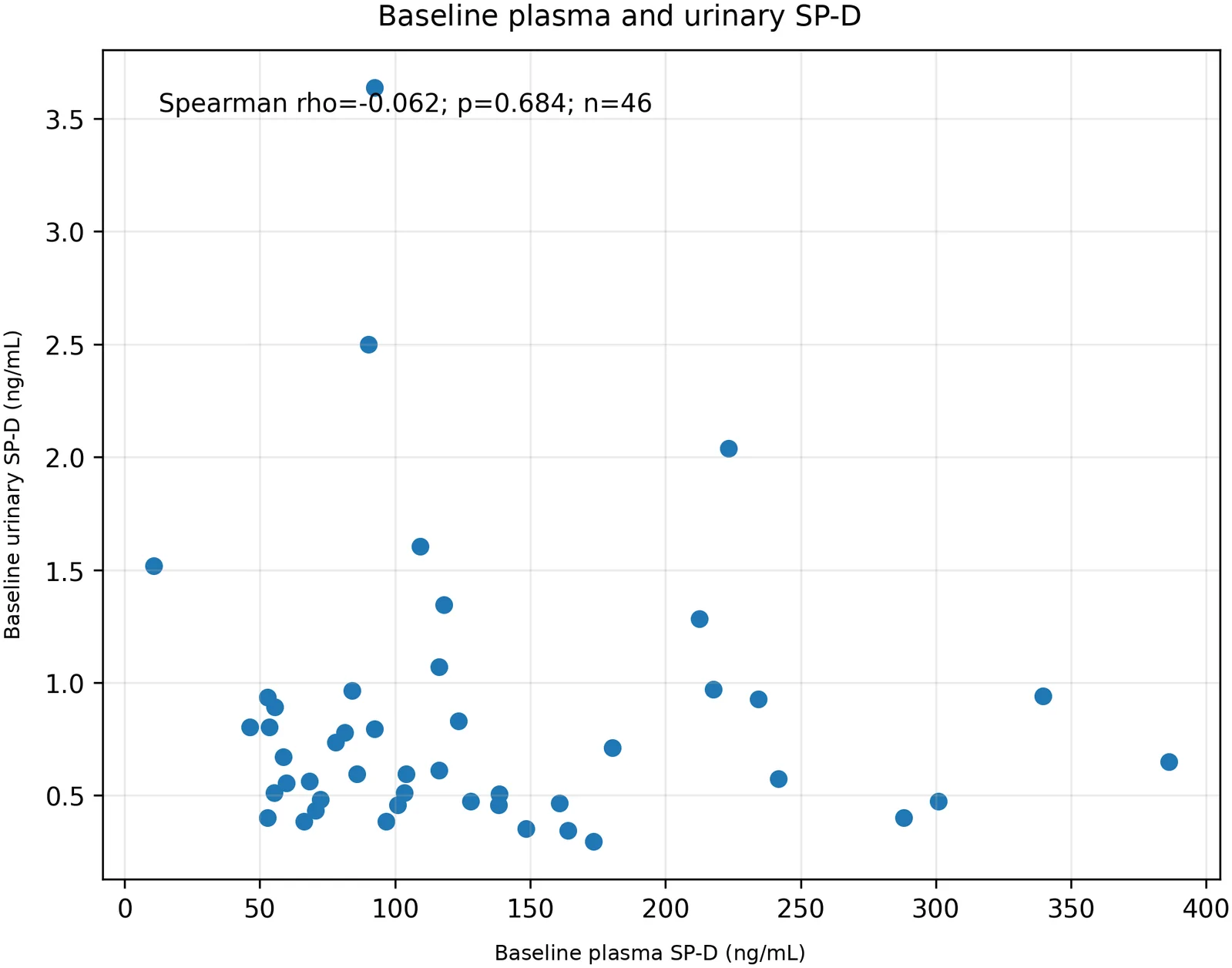
Baseline plasma and urinary SP-D concentrations; no inter-compartment correlation was observed.

**Figure 7:**
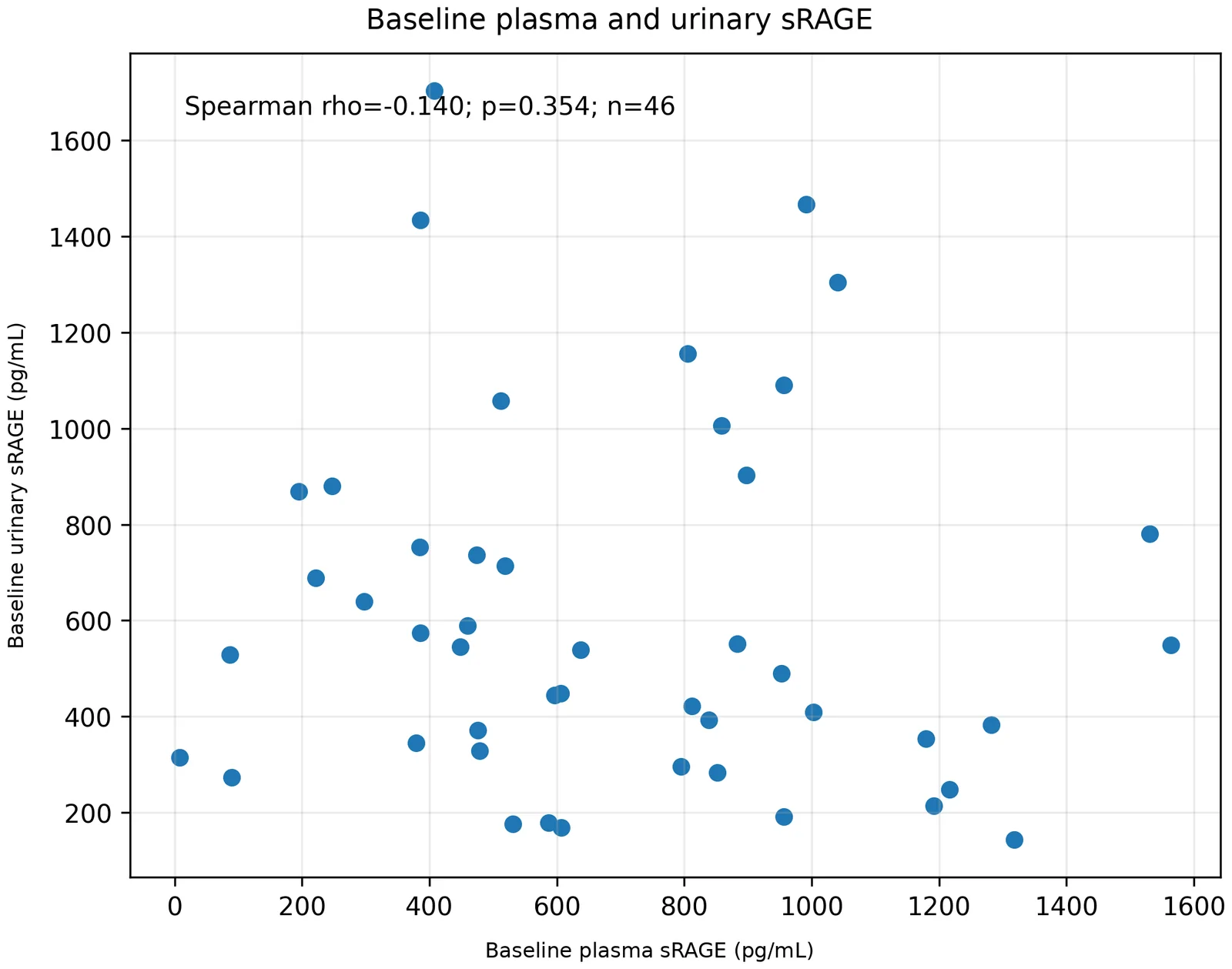
Baseline plasma and urinary sRAGE concentrations; no inter-compartment correlation was observed.

Baseline plasma and urinary SP-D were not correlated (rho=−0.062; p=0.684), nor were plasma and urinary sRAGE (rho=−0.140; p=0.354) (**Figures 6**, **7**). Across paired time points, SP-D showed a weak negative association (rho=−0.233; p=0.049; q=0.394; clustered beta=−0.200; p=0.046; R²=0.059). The absence of correlation between compartments at baseline suggests that plasma and urinary SP-D and sRAGE may capture partly distinct biomarker dynamics; the underlying mechanisms cannot be determined from the present data. Plasma SP-D primarily reflects alveolar epithelial leakage into the systemic circulation, whereas urinary SP-D may be shaped by renal tubular handling and local glomerular dynamics. The nominally significant negative longitudinal association between plasma and urinary SP-D across time points is of uncertain biological significance after multiple testing correction, but may reflect opposing dynamics: rising plasma SP-D during alveolar injury occurring concurrently with decreasing urinary SP-D concentrations. These findings indicate that plasma and urinary concentrations are not interchangeable and each compartment requires independent evaluation.

### 3.9 Separate adjuvant 1×BEP cohort

The separate adjuvant cohort comprised 18 patients; ten patients had paired plasma biomarkers. The adjuvant cohort consisted exclusively of Stage I germ cell tumour patients receiving single-cycle BEP as adjuvant therapy following radical orchiectomy, with no clinical or radiological evidence of residual disease at treatment initiation. By the definition of adjuvant therapy in this setting, these patients had no measurable tumour burden and no pulmonary metastases, thus eliminating tumour-related SP-D alterations and metastasis regression as potential confounding factors. To minimise confusion arising from the fundamentally different clinical contexts of adjuvant and therapeutic BEP, the two cohorts were deliberately analysed as separate subgroups throughout this study. Baseline plasma SP-D concentrations did not differ significantly between the adjuvant and therapeutic cohorts (median 83.6 vs 92.4 ng/mL; p=0.734), supporting comparability of pre-treatment biomarker levels. Median SP-D changed by −6.35% (p=1.000) and sRAGE by −2.82% (p=0.625). Only three patients had paired urinary measurements. The adjuvant cohort received a substantially lower cumulative bleomycin dose (90 mg, single cycle) compared with 270–360 mg in the therapeutic cohort. The absence of significant biomarker changes may be consistent with the lower cumulative treatment exposure in the adjuvant setting. The very limited number of paired urinary specimens (n=3) precludes any meaningful assessment of urinary dynamics in this subgroup. All findings in the adjuvant cohort should therefore be regarded as exploratory. The absence of a detectable increase in the small adjuvant cohort is compatible with a dose-related hypothesis, which requires prospective confirmation.

### 3.10 Comparison of 3×BEP and 4×BEP

Among patients with a cycle count, documented pulmonary toxicity occurred in 8 of 45 evaluable patients receiving three BEP cycles (17.8%) and in 5 of 11 patients receiving four cycles (45.5%). Although pulmonary toxicity was numerically more frequent after four BEP cycles, the difference was not statistically significant (OR 3.85; 95% CI 0.94–15.81; Fisher’s exact test p=0.104). Baseline plasma SP-D, maximum plasma SP-D, and last available percentage SP-D change did not differ significantly between the three- and four-cycle groups (p=0.255; p=0.726; p=0.660). Neutropenia and/or febrile neutropenia occurred in 13 of 46 patients receiving three cycles (28.3%) and in 1 of 11 patients receiving four cycles (9.1%; Fisher’s exact test p=0.261). Baseline plasma sRAGE, maximum plasma sRAGE, and last available percentage sRAGE change were not significantly higher in the four-cycle group (p=0.208; p=0.160; p=0.844). Maximum plasma sRAGE remained independently associated with haematological toxicity after accounting for three versus four BEP cycles (OR per doubling 8.24; 95% CI 1.74–38.99; p=0.0079).

## 4 Discussion

This exploratory translational study yields several statistically significant and clinically relevant findings. Concerning plasma SP-D dynamics during BEP chemotherapy, two conceptually distinct phenomena require careful disambiguation. First, plasma SP-D increased significantly over the course of treatment across the entire patient cohort (Friedman test p=0.00018; n=30 with complete measurements): this significant cohort-level increase is consistent with bleomycin-associated cumulative alveolar epithelial injury, although the present observational study cannot establish a specific causal class effect of bleomycin and individual SP-D trajectories varied. This pattern is biologically plausible given that SP-D is produced predominantly by type II alveolar cells and its plasma concentrations reflect the intensity of alveolar damage (7–9). Second, and independently, the amplitude of the SP-D rise was substantially greater in patients who developed HRCT-documented pulmonary toxicity than in those who did not: the last available percentage SP-D increase discriminated HRCT-positive from HRCT-negative patients (Mann–Whitney p=0.010; AUC 0.827, 95% CI 0.637–0.967; Youden threshold >79.7%: sensitivity 85.7%, specificity 84.2%). Crucially, the time × toxicity group interaction was not statistically significant, indicating that no statistically significant evidence of a difference in temporal SP-D trajectories between HRCT-positive and HRCT-negative patients was detected; the discriminatory information is carried by the overall amplitude of elevation rather than by a distinct temporal pattern or differential rate of rise. The amplitude difference did not survive multiple-testing correction (FDR q=0.228), appropriately designating this as an exploratory finding that requires prospective confirmation in an adequately powered, pre-specified study.

Higher baseline urinary SP-D was significantly associated with lower post-treatment DLCO (rho=−0.609; p=0.001; FDR q=0.014), representing, to the best of our knowledge, the first such report in a population of patients with germ cell tumours receiving BEP chemotherapy. Whether baseline urinary SP-D provides prognostic information about post-treatment pulmonary function independently of plasma SP-D concentration cannot be determined from the present data, as no model adjusting for plasma SP-D was performed. Interpretation of absolute urinary concentrations is, however, limited by the absence of normalisation to urinary creatinine; hydration status, urine concentration, and renal tubular handling may affect the results (1, 17).

Maximum plasma sRAGE was statistically significantly associated with neutropenia and/or febrile neutropenia during BEP chemotherapy (OR per doubling 12.13; 95% CI 2.59–56.93; p=0.0016; FDR q=0.013), with this association remaining robust after correction for multiple testing and after adjustment for the number of BEP cycles. Notably, baseline plasma sRAGE correlated with creatinine levels (rho=0.455; p=0.0028; FDR q=0.036) and negatively with eGFR (rho=−0.396; p=0.010), suggesting that renal function may influence circulating sRAGE concentrations; this dependence must be considered when interpreting sRAGE concentrations in populations with renal insufficiency or receiving nephrotoxic agents such as platinum derivatives.

In contrast, baseline plasma SP-D and sRAGE did not demonstrate predictive value for the development of documented pulmonary toxicity (OR 0.60 [95% CI 0.27–1.37]; p=0.230 and OR 1.20 [95% CI 0.55–2.62]; p=0.640, respectively). A biomarker used prior to treatment to guide individualised decisions on bleomycin inclusion would require a stronger, reproducible, and independently validated association with the relevant clinical endpoints (15, 16). Based on these exploratory data, no threshold baseline concentration can be recommended for clinical decision-making.

The differential effect of pulmonary metastases on the longitudinal trajectories of plasma SP-D (time × metastases interaction: p=0.012; adjusted p=0.027) is particularly interesting. SP-D increased less over time in patients with pulmonary metastases, consistent with the hypothesis that regression of metastatic lung deposits under chemotherapy may partially attenuate the bleomycin-driven SP-D rise by restoring alveolar barrier integrity in the affected parenchyma. An important consideration is whether this metastasis-related attenuation of SP-D kinetics might reduce the sensitivity or interpretability of SP-D as a biomarker of pulmonary toxicity in patients with pulmonary metastases. Stratified analyses address this directly and yield a counterintuitive but biologically coherent finding: among the seven patients with pulmonary metastases, both who developed HRCT-defined toxicity showed the largest SP-D increases in the entire cohort (SP-D increases of +91.0% and +216.6%), substantially exceeding all five HRCT-negative patients in that stratum (median +7.6%). Far from reducing biomarker sensitivity, the metastasis-related attenuation of the SP-D background signal in HRCT-negative patients appears to produce a more favourable signal-to-noise ratio in the metastasis subgroup: the HRCT-negative baseline is lower (because regressing metastases attenuate SP-D), while the toxicity-driven SP-D surge in HRCT-positive patients remains high, preserving discriminatory capacity. These results suggest that the metastasis × SP-D interaction reflects a clinically important contextual modifier of SP-D kinetics rather than a confounding source of false negatives, and that the utility of longitudinal SP-D monitoring is maintained regardless of pulmonary metastasis status. These data are exploratory and based on very small subgroups; prospective validation in a larger cohort with systematic HRCT assessment is warranted.

Low inter-compartment concordance between plasma and urinary concentrations of both SP-D and sRAGE suggests kinetics and regulation specific to individual biological matrices. Plasma and urinary values are therefore not interchangeable and cannot substitute for one another without prior independent validation (10, 11).

Strengths of the study include its prospective design with repeated sample collection, simultaneous assessment of plasma and urine, stratification of therapeutic and adjuvant cohorts, patient-level analysis, and control of the false discovery rate using the Benjamini–Hochberg method. Limitations include the small number of pulmonary toxicity events, missing and irregular measurement time points, the absence of centrally blinded assessment of pulmonary toxicity, potential centre effect, sparse urinary sampling, and the absence of normalisation to urinary creatinine. These results generate specific hypotheses for prospective biomarker studies and do not constitute an immediately clinically implementable test.

## 5 Conclusions

Plasma SP-D dynamics during BEP chemotherapy reflect two conceptually distinct biological phenomena that must be carefully distinguished in clinical interpretation. A significant cohort-level increase in plasma SP-D was observed across the entire cohort, irrespective of the development of HRCT-documented toxicity, consistent with bleomycin-associated cumulative alveolar epithelial injury. Separately, the amplitude of this rise was substantially greater in patients who developed HRCT-documented pulmonary toxicity, providing discriminatory capacity for identifying clinically significant bleomycin-induced injury; this discriminatory finding did not survive correction for multiple comparisons and should be regarded as exploratory, requiring prospective replication in an adequately powered, pre-specified study. Higher baseline urinary SP-D was associated with lower post-treatment diffusing capacity of the lungs — the finding most robustly supported after correction for multiple testing — highlighting the complementary value of the urinary compartment in capturing functional pulmonary sequelae. Maximum plasma sRAGE was strongly associated with neutropenia and/or febrile neutropenia during BEP chemotherapy, indicating that SP-D and sRAGE capture distinct dimensions of BEP toxicity. To the best of our knowledge, this is the first longitudinal biomarker study to characterise the dynamics of SP-D and sRAGE simultaneously across plasma and urinary compartments in patients receiving BEP chemotherapy for germ cell tumours, and these findings generate specific, testable hypotheses for prospective multicentre validation.

### 5.1 Clinical implications

- The current exploratory analysis supports the potential clinical relevance of longitudinal SP-D monitoring during BEP chemotherapy, while acknowledging that formal clinical utility — defined as the capacity to guide prospective treatment decisions using independently validated, pre-specified thresholds — requires confirmation in an adequately powered prospective study. The best available evidence from this exploratory dataset can be summarised as follows. First, longitudinal plasma SP-D discriminated HRCT-defined pulmonary toxicity with an AUC of 0.827 (95% CI 0.637–0.967; n=26); at the *post-hoc* Youden-optimal threshold of >79.7% increase from baseline, sensitivity was 85.7% and specificity 84.2%, at this *post-hoc* threshold, sensitivity was 85.7% in the present exploratory dataset. Second, baseline urinary SP-D was associated with lower post-treatment DLCO (rho=−0.609; p=0.001; FDR q=0.014), the only SP-D finding to survive multiple-testing correction, suggesting a role for pre-treatment urinary SP-D in identifying patients at greater risk of functional pulmonary impairment. Third, plasma and urinary SP-D capture complementary dimensions of bleomycin-induced damage — radiological structural injury (HRCT) and functional impairment (DLCO) — indicating that neither compartment is redundant and that whether combined plasma and urinary biomarker assessment improves clinical prediction requires prospective evaluation. It is important to note that the discriminatory performance of the SP-D amplitude difference did not survive multiple-testing correction (FDR q=0.228), and the *post-hoc* threshold cannot be used for clinical decision-making without prospective validation. Translated into a potential monitoring framework, serial plasma SP-D measurement during BEP chemotherapy — with prospectively defined and independently validated alert thresholds — could support early radiological re-evaluation in patients with the greatest SP-D amplitude increases, potentially enabling timely bleomycin dose modification before irreversible pulmonary fibrosis develops. The immune-mediated mechanism of bleomycin pulmonary toxicity — including dysregulated alveolar-immune crosstalk, innate immune activation, and profibrotic signalling — has recently been comprehensively reviewed in a comparative analysis of BEP, EP, and VIP regimens (6).
- The statistically significant association of maximum plasma sRAGE with neutropenia and/or febrile neutropenia (OR per doubling 12.13; 95% CI 2.59–56.93; p=0.0016) suggests a potential role for sRAGE as an integrated marker of systemic BEP toxicity; its clinical utility requires prospective validation with pre-specified threshold values and adjustment for renal function.
- A further prospective multicentre biomarker study in the germ cell tumour population is currently underway, specifically designed to address the key limitations of the present analysis and to provide the statistical power necessary for formal hypothesis testing. The study will incorporate central and blinded HRCT assessment, serial quantitative assessment of pulmonary tumour burden, normalisation of urinary biomarkers to creatinine, and primary analyses using prospectively defined threshold values and pre-specified hypotheses — including the distinction between the cohort-level SP-D increase observed during BEP and the amplitude difference associated with HRCT-documented toxicity — as *post-hoc* exploratory threshold values cannot serve as a basis for clinical decision-making. The multicentre prospective design is intended to provide the independent validation required to translate these exploratory findings into a clinically implementable biomarker-guided monitoring strategy.

## Data Availability

The raw data supporting the conclusions of this article will be made available by the authors, without undue reservation.
