# Supplementary material for: Longitudinal plasma and urinary SP-D and sRAGE during BEP chemotherapy in germ cell tumours
Source: Front Immunol. 2026 Sep 3;17:1954888. doi: 10.3389/fimmu.2026.1954888 (PMC13582606; doi:10.3389/fimmu.2026.1954888)
Supplement: Supplementary file 1 [file Table1.docx]

Supplementary Table S1. Characteristics of the entire therapeutic BEP cohort.

| Characteristic | Entire therapeutic cohort (n=60) | **3xBEP (n=46)** | **4xBEP (n=11)** |
| --- | --- | --- | --- |
| Age, years | 36.0 (32.0-41.0), n=59 | 37.0 (32.0-41.0), n=46 | 34.0 (24.0-36.5), n=11 |
| Age ≥30 years | 46/59 (78.0%) | 37/46 (80.4%) | 7/11 (63.6%) |
| Histology: seminoma | 25/58 (43.1%) | 21/46 (45.7%) | 2/10 (20.0%) |
| Histology: NSGCT/mixed tumour | 33/58 (56.9%) | 25/46 (54.3%) | 8/10 (80.0%) |
| Primary location: gonadal | 54/58 (93.1%) | 44/46 (95.7%) | 9/11 (81.8%) |
| Primary location: extragonadal retroperitoneal | 2/58 (3.4%) | 1/46 (2.2%) | 1/11 (9.1%) |
| Primary location: mediastinal | 1/58 (1.7%) | 1/46 (2.2%) | 0/11 (0.0%) |
| Stage I | 4/57 (7.0%) | 4/46 (8.7%) | 0/11 (0.0%) |
| Stage II | 26/57 (45.6%) | 26/46 (56.5%) | 0/11 (0.0%) |
| Stage III | 24/57 (42.1%) | 14/46 (30.4%) | 10/11 (90.9%) |
| IGCCCG: good prognosis | 31/42 (73.8%) | 30/31 (96.8%) | 1/11 (9.1%) |
| IGCCCG: intermediate prognosis | 6/42 (14.3%) | 1/31 (3.2%) | 5/11 (45.5%) |
| IGCCCG: poor prognosis | 5/42 (11.9%) | 0/31 (0.0%) | 5/11 (45.5%) |
| Pulmonary metastases | 12/55 (21.8%) | 9/44 (20.5%) | 3/11 (27.3%) |
| Smoking history | 28/56 (50.0%) | 25/45 (55.6%) | 3/11 (27.3%) |
| Haemoglobin, g/L | 150.0 (139.0-156.0), n=57 | 151.0 (141.2-156.8), n=46 | 140.0 (117.0-151.0), n=11 |
| Creatinine, µmol/L | 80.3 (73.9-91.5), n=56 | 82.0 (76.8-92.9), n=45 | 68.4 (61.4-81.5), n=11 |
| eGFR, mL/min | 108.9 (90.5-116.2), n=56 | 108.0 (88.2-112.8), n=45 | 125.4 (105.0-131.4), n=11 |
| Baseline DLCO, % predicted | 95.0 (85.5-101.5), n=55 | 97.0 (88.0-104.2), n=46 | 90.0 (79.0-95.0), n=9 |
| Post-treatment DLCO, % predicted | 96.0 (83.2-103.2), n=36 | 96.0 (86.0-103.5), n=27 | 96.0 (64.0-103.0), n=9 |
| DLCO change, percentage points | -4.0 (-12.8 to 2.5), n=34 | -4.0 (-11.5 to 2.0), n=27 | -4.0 (-26.5 to 4.0), n=7 |
| NLR | 2.47 (1.88-3.80), n=51 | 2.22 (1.77-3.28), n=40 | 3.63 (3.04-4.61), n=11 |
| PLR | 141.2 (115.0-210.3), n=51 | 140.3 (114.7-180.8), n=40 | 205.6 (158.9-255.9), n=11 |
| SII | 570.6 (404.5-885.6), n=51 | 547.3 (391.0-834.9), n=40 | 872.1 (472.3-1117.1), n=11 |
| Cumulative bleomycin dose, mg | 270 (270-360), n=39 | 270 (270-270), n=28 | 360 (360-360), n=11 |
| Documented pulmonary toxicity | 13/59 (22.0%) | 8/45 (17.8%) | 5/12 (41.7%) |
| Neutropenia and/or febrile neutropenia | 14/57 (24.6%) | 13/46 (28.3%) | 1/11 (9.1%) |
| Favourable treatment response | 36/39 (92.3%) | 28/28 (100.0%) | 8/11 (72.7%) |

The Entire therapeutic cohort column includes all 60 patients. Patients with three and four BEP cycles are retained in separate columns. Data are presented as median (interquartile range), n or n/N (%) with denominators according to available data. NSGCT, non-seminomatous germ cell tumour; IGCCCG, International Germ Cell Cancer Collaborative Group; DLCO, diffusing capacity of the lung for carbon monoxide.
